# Supplementary material for: Rheological Investigation of Relaxation Behavior of Polycarbonate/Acrylonitrile-Butadiene-Styrene Blends
Source: Polymers (Basel). 2020 Aug 25;12(9):1916. doi: 10.3390/polym12091916 (PMC7563493; doi:10.3390/polym12091916)
Supplement: Supplementary file 1 [file polymers-12-01916-s001.pdf]

Supplementary Information

Rheological Investigation of Relaxation Behavior of Polycarbonate/Acrylonitrile-Butadiene-Styrene Blends

Jae Sik Seo[1,2], Ho Tak Jeon[2,*], Tae Hee Han[1,*]

[1] Department of Organic and Nano Engineering, Hanyang University, Seoul 04763, Republic of Korea; than@hanyang.ac.kr

[2] Interior System Plastic Materials Development Team, Material Development Center, Hyundai Motor Company, Hwaseong 18280, Republic of Korea; htjeon@hyundai.com

* Correspondence: than@hanyang.ac.kr; Tel: +82-2-22200493

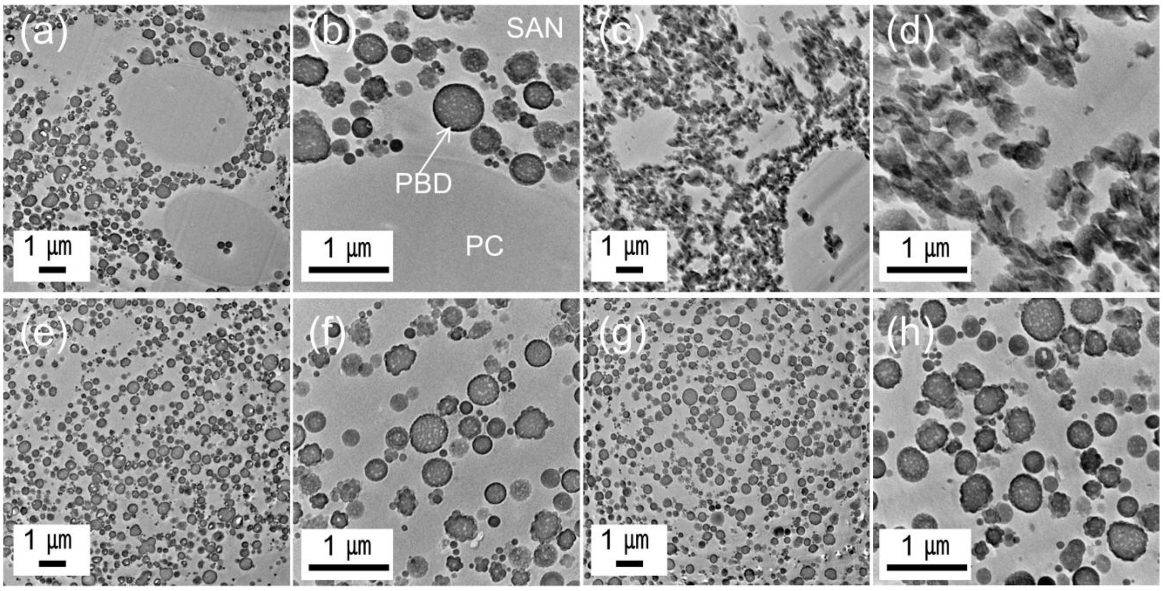

Figure S1. The phase change of PC/ABS blends examined by TEM for the blend ratio of (a-d)5:5 and (e-h) 0:10; quenched to the room temperate right after time sweep measurement with the frequency of (a-b) 0.1 rad/s, (c-d) 1 rad/s, and the images of (e-h) are obtained from the same corresponding conditions.

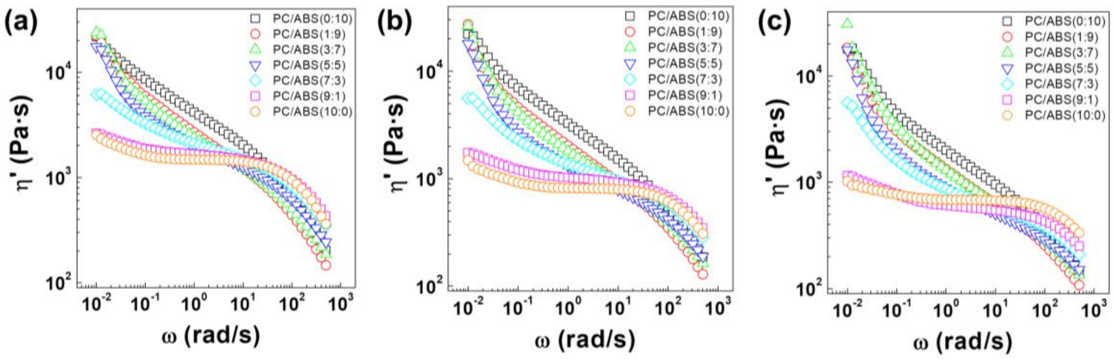

Figure S2. Viscosity curves of PC/ABS blends at the measuring temperature of (a) 240 °C, (b) 250 °C, and (c) 260 °C.

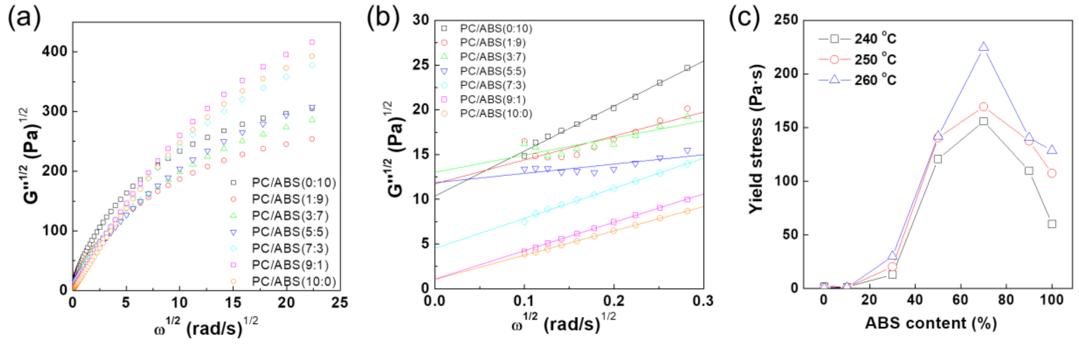

Figure S3. The yield stress behavior of PC/ABS blends; (a) Casson plot of the blends with whole frequency range at 250 °C, (b) difference in intercepts of the blends, (c) change of yield stress values of the blends with increasing ABS content at given temperatures.
